# Massive pulmonary embolism after cesarean section in a pregnant with occult pneumonia after COVID-19 infection: A case report

**DOI:** 10.1097/MD.0000000000042327

**Published:** 2025-05-09

**Authors:** Huijun Ye, Yi Yang, Ping Zhu, Huiling Zheng, Yunxia Lin, Jiali Liu, Ruilan Li, Lihua Jin

**Affiliations:** a Department of Gynaecology and Obstetrics, The Second Affiliated Hospital of Zhejiang Chinese Medical University, Hangzhou, China; b Radiology Department, The Second Affiliated Hospital of Zhejiang Chinese Medical University, Hangzhou, China.

**Keywords:** case report, COVID-19 infection, cryptogenic pneumonia, CTPA, post-cesarean section, pregnant women, pulmonary embolism

## Abstract

**Rationale:**

Pregnant and postpartum women have a hypercoagulable state, and COVID-19 infection further heightens the risk of venous thromboembolism, with pulmonary embolism (PE) in COVID-19-infected pregnant women being a global concern.

**Patient concerns:**

A 47-year-old pregnant woman with a history of 3 vaginal deliveries and 1 cesarean section, who had a neo-coronavirus infection 29 days ago with rapid symptom resolution, developed asymptomatic massive PE within 24 hours after cesarean section at 38+ weeks of gestation.

**Diagnoses:**

Massive PE was diagnosed by computed tomography pulmonary angiography, which showed thromboembolism in multiple parts of the pulmonary arteries, along with pleural effusion and lung inflammation. Cardiac ultrasound revealed mildly elevated pulmonary artery systolic pressure and mild mitral and tricuspid regurgitation.

**Interventions:**

Anticoagulation therapy was initially with enoxaparin sodium injection, and the dosage was adjusted. Intravenous anti-infection therapy was also given. After discharge, the patient was switched to rivaroxaban. Compression stockings were used for venous thromboembolism prevention.

**Outcomes:**

After 5 months of anticoagulation therapy, the computed tomography pulmonary angiography of the pulmonary artery was completely normalized, but a small amount of interstitial lung lesions still persisted.

**Lessons:**

Pregnant women infected with the new coronavirus need close attention during pregnancy and puerperium, especially those with thrombosis risk factors who should receive early anticoagulation. There are many aspects worthy of further exploration, such as the necessity of early pulmonary computed tomography scans and appropriate anticoagulant dosage in pregnant women with COVID-19 infection.

## 1. Introduction

Venous thromboembolism (VTE) includes pulmonary embolism (PE) and deep vein thrombosis (DVT). It is crucial to emphasize that women’s bodies undergo significant hypercoagulation during pregnancy and the postpartum period. Consequently, the incidence of VTE significantly increases during this time, with reports indicating that pregnant women face a 4 to 5 times higher risk of VTE compared to nonpregnant women.^[1]^ The viral pneumonia epidemic that emerged at the end of 2019 and spread rapidly around the world was named coronavirus disease (COVID-19). It is worth highlighting that the risk of VTE escalates significantly in pregnant and postpartum individuals infected with COVID-19. This heightened risk primarily stems from the potent inflammatory response triggered by COVID-19 infection, characterized by the release of cytokines, chemokines, and cellular activation. These processes create a hypercoagulable state through the intricate interplay between inflammation, complement activation, and coagulation.^[2]^ Therefore, once the COVID-19 virus infects a pregnant woman, it causes a massive inflammatory response, which will further increase the risk of VTE complications in pregnant women who are already in a hypercoagulable state.^[1]^ PE in pregnant women infected with COVID-19 has become a global concern,^[3]^ which can indirectly increase the risk of death due to COVID-19.

## 2. Consent

This study was approved by the ethics committee of the Second Affiliated Hospital of Zhejiang Chinese Medical University. Patient has provided informed consent for publication of the case.

## 3. Case report

We next report a case of a pregnant woman who developed a massive PE 29 days after neo-coronavirus infection and 1 day after cesarean section:

Chinese female, 47 years old, 4-0-0-4, with a history of 3 vaginal deliveries and 1 cesarean section, experienced a bout of illness 29 days ago December 21, 2022. She was infected by a new coronavirus and had a fever, reaching a peak temperature of 39.5°C. Her symptoms included a sore throat, cough with sputum production, malaise, shortness of breath, dizziness, and a positive test result for the nucleic acid of the new coronavirus. Acetaminophen was given 1 tablet and Chinese medicine is taken orally twice a day, and on the next day the temperature was normalized, and shortness of breath, fatigue, and dizziness disappeared.

Three days later, all clinical symptoms had vanished: there was no longer any coughing, sputum production, sore throat, fatigue, fever, shortness of breath, or other discomforting symptoms. No further medical intervention was deemed necessary at that point. At the time, a CT scan of the lungs was not conducted, and the patient did not experience any subsequent pregnancy-related discomfort. Routine prenatal examinations revealed no abnormalities.

She underwent cervical loop electrosurgical excision procedure 1 year ago for cervical intraepithelial neoplasia grade 2, she has no other medical history and no known allergies, and she does not smoke or drink alcohol and has no relevant family history. She was 160 cm tall, weighed 58 kg, and had a body mass index: 22.66. Her antepartum examination showed no obvious abnormalities.

On January 19, 2023, electronic monitoring of fetal heart rate suggests severe variable deceleration and the fetus cannot be delivered vaginally within a short period of time, the patient underwent a lower uterine segment cesarean section at 38+ weeks of gestation due to “intrauterine distress.” The operation went smoothly with minimal intraoperative bleeding, and he was returned to the ward after the operation with electrocardiographic monitoring, oximetry monitoring, and low-flow oxygen intake by a routine nasal cannula, and perioperative management was performed according to the enhanced recovery after cesarean process. Because the patient’s postoperative venous thrombosis was assessed as high-risk (age ≥ 35 years old, parity ≥ 3 times, emergency cesarean section, caprini Scale and VTE Risk Assessment in Pregnancy and Puerperium Scale: 4), the patients had received compression stockings postoperatively. Twelve hours later, subcutaneous anticoagulation with enoxaparin sodium injection (4000 AxaIU qd) was initiated, and there were no complaints of dizziness, headache, chest tightness, palpitations, shortness of breath, chest pain, cough, sputum, or any other discomforts. Twenty-four hours after the operation January 20, 2023, when the nurse was ready to give the patient to stop nasal catheter oxygen intake, she observed that the patient’s blood oxygen saturation (SpO_2_) in the absence of oxygen intake fell to 92% to 94%, and in the state of low-flow oxygen intake oxygen saturation was 95% to 98%, and there was still no cyanosis of lips and mouth, chest tightness, chest pain, shortness of breath, cough and cough, nausea and vomiting, dizziness, and other discomforts. Physical examination revealed a body temperature of 36.7°C, blood pressure 132/75 mm Hg, clear consciousness, absence of cyanosis in the lips and nails of the limbs, normal cardiopulmonary auscultation, a heart rate of 72 beats/min with normal rhythm, absence of dry or wet rales in the lungs, a soft abdomen without rebound pain, good uterine contraction, minimal vaginal bleeding, and normal dorsalis pedis artery pulsation. The patient had no edema of the lower extremities, no sensory abnormalities such as pain or numbness, no skin cyanosis, dorsalis pedis arterial pulsations were present, and Homans and Bauer’s signs were negative.

**Figure 1.**
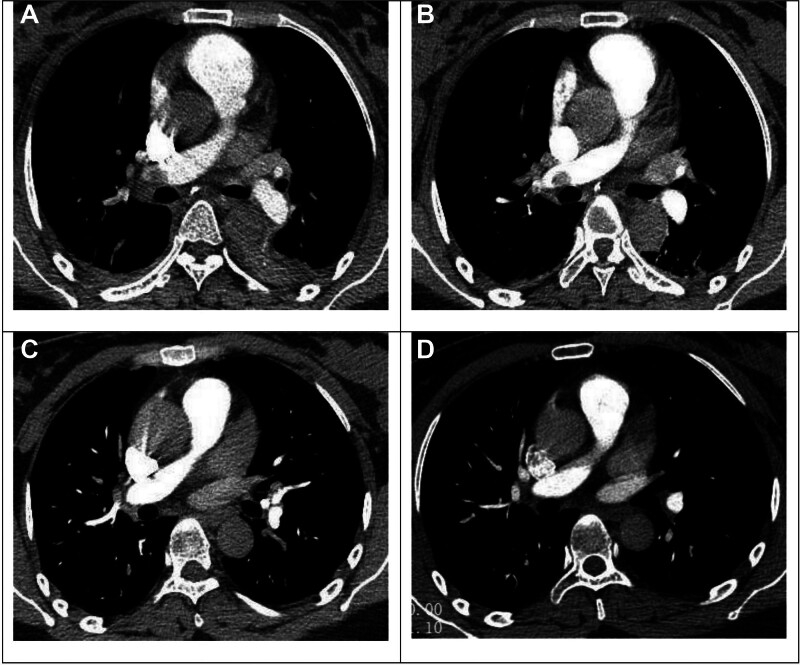
CTPA imaging presentation. (A) A shadow of a filling defect was seen in the lumen of the main trunk of the right lower pulmonary artery and the primary and secondary major branches, and thromboembolic formation was considered. (B) Thromboembolism formation in the main trunk of the right lower pulmonary artery and the lumen of the primary and secondary major branches, which was better than Figure A. (C) A little thromboembolism in the lumen of the primary and secondary major branches of the right lower pulmonary artery, significantly better than Figure B. (D) CTPA showed no obvious abnormality. CTPA = computed tomography pulmonary angiography.

**Figure 2.**
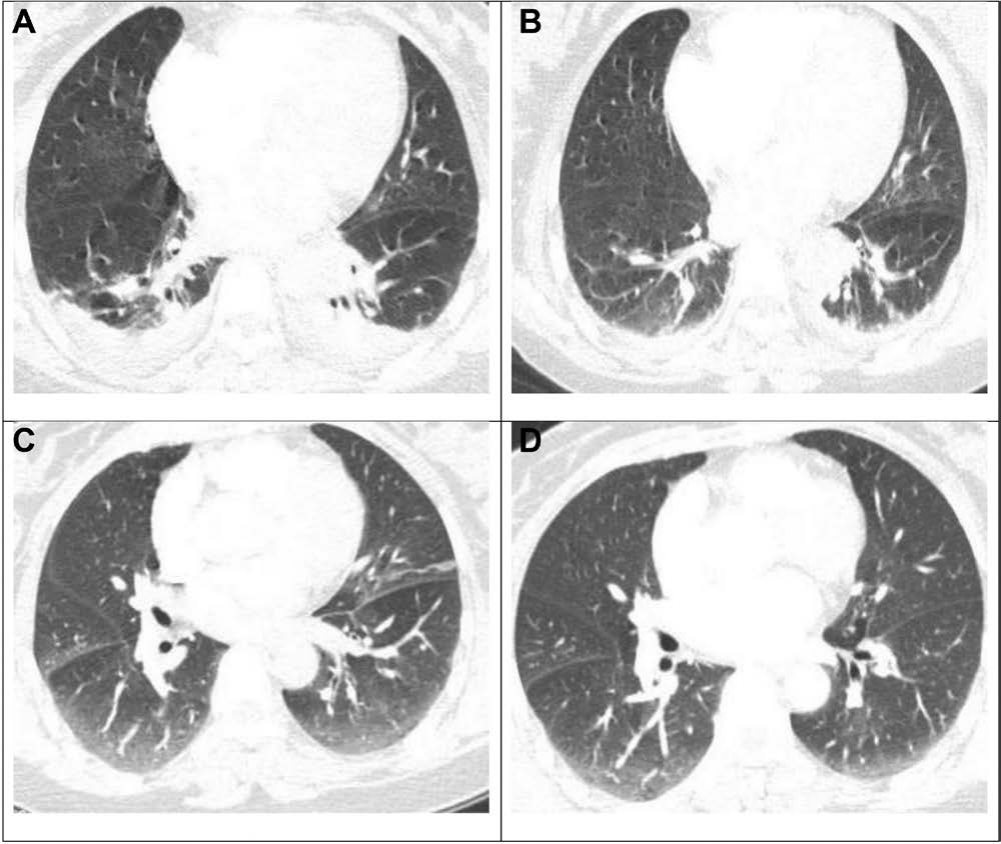
CT plain lung window of the lungs. (A) A small amount of pleural effusion on both sides with mild distensibility of the lower lobes of both lungs; and a little inflammation of the lower lobes of both lungs. (B) A small amount of pleural effusion on both sides with mild distensibility of the lower lobes of both lungs; a little inflammation of the lower lobes of both lungs; better than Figure A. (C) A few interstitial changes in the lower lobes of both lungs. (D) Few interstitial changes in the lower lobes of both lungs; better than Figure C.

Auxiliary examination: January 20, 2023 blood routine: white blood cell (WBC): 8.2 × 10^9^/L, neutrophil percentage (NE%): 81.9%, hemoglobin: 113 g/L, platelet count (PLT): 112 × 10^9^/L. Coagulation function routine: thrombin time (TT): 13.4 s, fibrinogen (FIB): 4.30 g/L, D2 polymer: 6.85 mg/L. (Normal value range: WBC: 3.5–9.5 × 10^9^/L, N%: 40.0–75.0%, L%: 20.0–50.0%, PLT: 125–350 × 10^9^/L, C-reactive Protein [CRP]: 0.0–8.0 mg/L. PT: 9.2–13.9 s, APTT: 21.2–34.8 s, D2 polymer: 0–0.55 mg/L, FIB: 2.0–4.0 g/L). Computed tomograph pulmonary angiography (CTPA) revealed localized thromboembolism in the apical and anterior segments of the upper lobe of the right lung, the lateral segments of the middle lobe, the main trunk of the right lower pulmonary artery, and branches at all levels of the lower lobe. Additionally, there was a small amount of pleural effusion on both sides accompanied by mild distension insufficiency in the lower lobes of both lungs, as well as slight inflammation in the lower lobes of both lungs. Cardiac ultrasound: mildly elevated pulmonary artery systolic pressure (pulmonary artery systolic pressure 45 mm Hg), left ventricular ejection fraction >55%, mild mitral and tricuspid regurgitation. Vascular ultrasound of the carotid artery, jugular vein, both upper limbs, and both lower limbs revealed blood flow in bilateral internal carotid arteries and veins, and bilateral upper limb veins, while both lower limb deep veins showed smooth blood flow. After a multidisciplinary consultation in the hospital, the patient’s anticoagulant therapy was modified to enoxaparin sodium injection (4000 AxaIU q12h) subcutaneously, and intravenous anti-infection therapy was initiated (Figs. 1A and 2A).

January 21, 2023 Blood routine: WBC: 8.8 × 10^9^/L, neutrophil percentage: 92%, hemoglobin: 123 g/L, PLT: 138 × 10^9^/L, CRP: 85.88 mg/L. Coagulation function routine: TT: 15.3 s, FIB: 6.20 g/L, D2 polymer: 1.52 mg/L. Anticoagulation and anti-infection treatment were continued. 2024-1-24 Review CTPA: Identified thromboembolism in the lumen of the main trunk of the right lower pulmonary artery and the major branches of the first and second levels, a small amount of pleural effusion on both sides accompanied by mild insufficiency of expansion of the lower lobes of the 2 lungs; a small amount of inflammation in the lower lobes of the 2 lungs. Throughout the hospitalization, no conscious symptoms such as chest tightness and shortness of breath were reported, and the same treatment as before was administered. (Figs. 1B and 2B). January 23, 2023 Review of blood routine: normal, CRP: normal. Coagulation function: TT:15.1 s, FIB:4.19 g/L, D2 polymer: 2.09 mg/L, stop antibiotics, continue anticoagulation, the patient was discharged on January 31st. After discharge, she was given rivaroxaban 15 mg orally twice a day, and breastfeeding was stopped. 3 weeks later, she was changed to 20 mg orally once a day for 6 months and was followed up for a long period by the cardiology department, respiratory medicine department, and vascular surgery department. February 8, 2023 D2 polymer: 0.67 mg/L. March 20, 2023 CTPA: a little thromboembolism in the lumen of the first and second major branches of the pulmonary artery in the lower lobe of the right lung. Few interstitial changes in the lower lobes of both lungs. (Figs. 1C and 2C). April 3, 2023 D2 polymer: 0.42 mg/L, June 12, 2023 Repeat CTPA of the pulmonary artery did not show any significant abnormality, with few interstitial changes in the lower lobes of both lungs (Figs. 1D and 2D).

## 4. Discussion

Pregnancy-related VTE accounts for approximately 10% of VTE in women and 9% of pregnancy-related deaths.^[4]^ VTE includes PE and DVT, and activation of the coagulation system, endothelial damage, and venous stasis all contribute to the increased risk during pregnancy,^[4]^ resulting in a 4- to 5-fold higher risk of VTE in pregnant women compared to nonpregnant women, with an incidence of 1.2% of every 1000 deliveries, and another 3- to 4-fold increase in the risk of VTE with cesarean delivery.^[5]^ After the onset of neo-coronavirus infection, studies have found an increased risk of venous and arterial thrombosis in pregnant women with COVID-19 compared to those without COVID-19 infection, and the severity of the disease and other traditional risk factors for pregnancy-related VTE may further increase this risk. However to date there is less information on the risk of postpartum thrombosis in COVID-19 pregnant women, and pregnant women have been systematically excluded from large randomized controlled trial studies.^[6]^

Every barber knows that COVID-19 is an infectious disease caused by a novel coronavirus, SARS-CoV-2, a single-stranded RNA virus that infects cells by binding to the angiotensin-converting enzyme-2 receptor. Infected patients may be asymptomatic or present with clinical signs such as fever, cough, sore throat, and headache, or they may develop a serious illness such as VTE. Although symptoms of acute COVID-19 infection usually last for 2 to 3 weeks, there is increasing evidence that SARS-CoV-2 may persist in multiple organ tissues of the human body after acute infection: viral protein and/or RNA and the virus may persist fora long time.^[7]^ As a result, 10% of patients develop persistent or new symptoms after the acute phase; this condition is referred to as Long COVID (sometimes referred to as post-acute sequelae of COVID-19), a multisystemic disease in which symptoms of Long COVID have been observed to persist for up to 3 years.^[7]^

Worldwide, there are at least 65 million individuals grappling with persistent coronaviral infections, with daily surges in cases.^[8]^ The disruption it inflicts upon the circulatory system encompasses endothelial dysfunction and its subsequent cascading effects, including an elevated susceptibility to DVT, PE, and hemorrhagic events. The mechanisms of viral factors (persistence, reactivation, and phagocytosis of SARS-CoV-2), host factors (chronic inflammation, metabolic and endocrine dysregulation, immune dysregulation, and autoimmunity), and downstream effects (initial infection-induced tissue damage, tissue hypoxia, host ecological dysregulation, and autonomic nervous system dysfunction) culminate in the development of an endothelial system characterized by thrombophilic endothelial inflammation, endothelial inflammation, overactivated platelets, and Fibrin-like micro-clots,^[7]^ leading to the development of VTE. VTE is classified into DVT and PE. Notably, PE is more prevalent during the puerperium period than DVT. Clinical manifestations of DVT typically include swelling and pain in the extremities or lower extremity regions, while PE presents with symptoms like dyspnea (labored breathing), chest pain, and hemoptysis (coughing up blood). In severe cases of PE, cyanosis (bluish discoloration, particularly of the lips and fingers) may occur, and an abundance of PEs can precipitate cardiac arrest within hours due to disseminated intravascular coagulation and subsequent multi-organ failure.^[9]^

Both pregnancy and COVID-19 infection amplify the risk of VTE: once the COVID-19 virus infects a pregnant woman, it causes a massive inflammatory response that further increases the risk of VTE.^[1]^ The estimated incidence of VTE during pregnancy and the postpartum period is 1 to 2 cases per 1000 deliveries, with the risk increasing notably during the postpartum phase, particularly following cesarean sections.^[10]^ Approximately 75% to 80% of pregnancy-related VTE cases are caused by DVT and 20% to 25% by PE. CTPA is the test of choice for evaluating patients with PE,^[11]^ The primary treatment for acute VTE in pregnancy and postpartum is anticoagulation.^[12]^ Enoxaparin, among these options, offers distinct advantages, including superior bioavailability, an extended half-life, more predictable anticoagulant effects, reduced bleeding risks, and a decreased likelihood of heparin-induced thrombocytopenia and bone loss.^[10]^ In 2011, rivaroxaban marked a milestone as the first direct oral anticoagulant endorsed by the European Medicines Agency for managing acute symptomatic DVT and for secondary prevention of recurrent VTE. In comparison to low molecular heparin, which necessitates subcutaneous injections, rivaroxaban tablets present a more convenient option for oral administration in an outpatient setting, thereby enhancing patient compliance. The patient was therefore switched to rivaroxaban tablets orally after discharge.

In this case, the patient contracted neo-coronavirus infection 29 days prior. While the associated clinical symptoms subsided after just 3 days, a severe PE manifested within 24 hours following a cesarean section. Subsequent imaging via CTPA revealed persistent pneumonitis and interstitial lung lesions. Consequently, we posit a potential correlation between the patient’s PE and neo-coronavirus growth. The International Society of Infectious Diseases in Obstetrics and Gynecology believes that, irrespective of other risk factors, all women diagnosed with COVID-19 should receive at least 10 days of low molecular weight heparin, preferably at an elevated dosage in cases of severe COVID-19 disease.^[3]^ The Swiss Society of Obstetrics and Gynecology believes that patients with COVID-19 are at high-risk of thromboembolism, which increases further with pregnancy and postpartum conditions, and that interdisciplinary thromboembolism prophylaxis should be offered to patients with COVID-19 during pregnancy and postpartum.^[3]^ However, it is worth noting that to date, no universally accepted global obstetric clinical guidelines or consensus have emerged regarding this matter.

For patients at high-risk of VTE, the clinical practice guidelines of the American College of Chest Physicians suggest that VTE should be prevented by a combination of mechanical methods based on pharmacologic anticoagulation,^[13]^ and mechanical methods include the use of graduated compression stockings, intermittent pneumatic compression (IPC), foot compression devices, and so on.^[13]^ IPC, and foot compression devices.^[13]^ Theoretically, the use of IPC has a better preventive effect, but graduated compression stockings is cheaper, more comfortable, and more convenient, and therefore it is the most commonly used mechanical preventive device in the clinic. In this case, we used compression stockings to prevent VTE, and now we are reflecting on whether the use of IPC during and after surgery would have a better preventive effect. Of course, the use of IPC has its contraindications and risks: contraindications include lower limb ischemia, skin or tissue necrosis or infection, significant lower limb swelling or pulmonary edema due to congestive heart failure, acute DVT, and malignant tumors of the extremities, and side effects are mainly local skin injuries. In the case of pregnant women, who often have lower extremity edema and obesity in late pregnancy, the use of IPC devices often feels uncomfortable and inconvenient.

The patient’s medical history reveals multiple risk factors for thrombosis: neo-coronavirus infection, advanced age (46 years), multiple births (3 vaginal deliveries and 1 cesarean delivery), and cesarean section, and she developed a severe PE despite having begun subcutaneous prophylaxis for thrombophilia with enoxaparin sodium needles 12 hours after the cesarean section according to the Thrombosis High-Risk Scale. This leads us to ponder the question: Should this patient have undergone a pulmonary CT scan and received low molecular weight heparin anticoagulation treatment when she initially contracted neo-coronavirus infection at 34 weeks gestation? Are there ethical considerations in conducting lung CT scans in pregnant women without evident signs of pneumonia? This necessitates a global consensus; Not all patients with PE display symptomatic, as seen in this case where the patient had a massive PE but remained asymptomatic, with only a slight decrease in finger-blood oxygen saturation monitoring on cessation of nasal cannula oxygenation. Therefore, it is crucial to be vigilant when observing a drop in oxygen saturation in high-risk patients, and CTPA should be promptly performed; Timely detection and treatment, coupled with multidisciplinary consultation, are pivotal in devising an individualized treatment plan that significantly impacts the patient’s prognosis. The consequences of failing to detect and treat this patient in a timely manner would have been catastrophic; Lung damage following neo-coronavirus infection in pregnant women may persist for an extended period. Even 5 months post-surgery and despite anti-infective treatment, the patient’s lung infection and interstitial lesions had not completely resolved. This condition could potentially affect cardiopulmonary function if not addressed promptly; When choosing physical prophylaxis, should all pregnant women at high-risk of thrombosis use the IPC device? However, this device is complicated to use and not very comfortable for the patient; Dosage of low molecular heparin. In this case, we used only a medium dose of heparin for fear of post-cesarean section incision and vaginal bleeding, should we have used the full amount of heparin? This is a question that deserves our introspection and thinking and correction.

## 5. Conclusion

In conclusion, adequate attention should be paid to pregnant women infected with new coronaviruses during pregnancy and puerperium. In particular, early anticoagulation should be given to patients with high-risk factors for thrombosis.

## Author contributions

**Conceptualization:** Ping Zhu.

**Data curation:** Huiling Zheng, Yunxia Lin.

**Investigation:** Jiali Liu, Ruilan Li.

**Validation:** Yi Yang.

**Writing – original draft:** Lihua Jin.

**Writing – review & editing:** Huijun Ye.
